# Assessing the Impact of Hypertension on Health-Related Quality of Life: Insights from Sociodemographic, Economic, and Clinical Features Using SF-36

**DOI:** 10.3390/healthcare13070838

**Published:** 2025-04-07

**Authors:** Geetha Kandasamy, Mona Almanasef, Khalid Orayj, Asma M. Alshahrani, Shada M. Alahmari

**Affiliations:** 1 Department of Clinical Pharmacy, College of Pharmacy, King Khalid University, Abha 61421, Saudi Arabia; 2 Department of Clinical Pharmacy, College of Pharmacy, Shaqra University, Dawadimi 11961, Saudi Arabia; 3 Pharmacy Services, Khamis Mushayt Maternity and Children Hospital, Abha 61961, Saudi Arabia

**Keywords:** hypertension, cross-sectional study, SF-36, HRQoL

## Abstract

**Background:** Hypertension significantly impacts the health-related quality of life (HRQoL) of patients. This study evaluates the influence of sociodemographic, economic, and clinical features on HRQoL among hypertensive patients using the 36-Item Short Form Survey (SF-36). **Method:** A cross-sectional study was conducted at a public health center in Khamis Mushayt, Saudi Arabia, where 209 adult hypertensive patients were surveyed using simple random sampling. Data were collected through a structured questionnaire covering sociodemographic and clinical details, and multiple linear regression was used to analyze the associations between variables and the SF-36 domains. Results: Of the 209 participants, 122 (58.4%) were female and 87 (41.6%) were male. Complications and multiple antihypertensive medications were linked to poorer physical functioning and general health (*p* < 0.05). Salt restriction improved physical functioning (B = 12.339, *p* = 0.008), and exercise reduced body pain (B = −8.487, *p* = 0.038). Middle-income patients had higher vitality (B = 7.632, *p* = 0.038) and social functioning (B = 16.465, *p* = 0.035). Higher-income individuals showed lower social functioning (B = −12.323, *p* = 0.022). **Conclusions:** Age, income, marital status, and complications were key determinants of HRQoL in hypertensive patients. Lifestyle interventions like exercise and salt restriction improve physical functioning and reduce pain, while psychological and social support are vital for mental health. Tailored interventions addressing clinical and psychosocial support are crucial for optimizing HRQoL in this population.

## 1. Introduction

Hypertension is the leading cause of mortality and disability worldwide [1], accounting for approximately 10.4 million deaths annually [2]. Despite significant advancements in medical technology, the prevalence of hypertension continues to rise at an alarming rate [3]. Previous studies project that by 2025, the global burden of hypertension will affect approximately two billion people [4,5]. Hypertension is a major contributor to cardiovascular disease, chronic kidney disease, and stroke, and is one of the leading causes of the global burden of non-communicable diseases [6]. According to the World Health Organization (WHO), quality of life is how individuals perceive their position in life, in relation to their goals, aspirations, standards, and concerns, as well as the culture and value systems in which they live [7]. In clinical and public health research, HRQoL is often described as a broad and multifaceted concept that typically reflects an individual’s physical and mental health status [8]. Additionally, HRQoL has been recognized in the literature as an important patient outcome for assessing the efficacy of interventions [9]. Various tools have been developed to accurately assess individuals’ HRQoL [10].

Over the past 15 years, HRQoL has gained increasing significance in clinical research [11]. It provides a comprehensive perspective that considers a patient’s social, emotional, and physical functioning [12]. A person’s physical and mental well-being, level of independence, and social relationships all have a complex impact on their HRQoL, which is influenced by how they perceive their place in life within the context of cultural and value systems [13]. Emotional and psychological factors are closely associated with hypertension, particularly during periods of high stress in life [14]. The effects of a single disease may be exacerbated by the presence of multiple comorbidities. Additionally, the negative impact of chronic illnesses on an individual’s quality of life can be influenced by factors such as age, gender, and socioeconomic status [15]. The rising mortality rates among middle-aged populations have underscored the need to link improvements in HRQoL with increased longevity. Some studies suggest that as life expectancy increases, a larger proportion of individuals experience poor health, which places greater strain on society and healthcare systems [15]. According to research by Wilson and Cleary, symptoms resulting from disease or treatment-induced physiological changes can impact functional status or HRQoL. Patient-related and environmental factors, which may influence how the patient perceives symptoms and changes in HRQoL, play a significant role in these correlations [16]. Hypertension is a leading cause of mortality and disability, with its rising prevalence straining healthcare systems. Understanding its impact on HRQoL is vital, as it affects physical, mental, emotional, and social well-being. Improving HRQoL will help individuals live not just longer lives but healthier ones.

This study builds on previous research by exploring the relationship between hypertension and HRQoL in the Abha Asir region of Saudi Arabia. It addresses gaps in understanding the influence of sociodemographic, economic, and cultural factors on HRQoL, and examines how age, gender, socioeconomic status, comorbidities, treatment adherence, and psychological stress affect HRQoL in hypertensive individuals, while evaluating the effectiveness of the SF-36 tool in diverse healthcare settings. Despite hypertension’s prevalence, its impact on HRQoL in this region remains underexplored, particularly regarding sociodemographic and cultural influences.

The primary hypothesis of the study is that hypertension negatively impacts HRQoL, with the extent of impact varying based on sociodemographic, economic, and clinical features as measured by the SF-36 scale. Therefore, the aim of the study is to assess the impact of hypertension on HRQoL by analyzing the influence of these factors using the SF-36 tool. This study enhances the understanding of hypertension’s impact on HRQoL by examining sociodemographic, economic, and clinical features using the SF-36 tool in Saudi populations. The findings will inform tailored healthcare policies, support personalized treatments, and suggest improvements to HRQoL assessment tools like the SF-36 for diverse settings.

## 2. Methods

### 2.1. Settings and Design

This study was an institution-based, cross-sectional research study conducted among adult hypertensive patients receiving treatment at a public health center in Khamis Mushayt from November 2024 to December 2024.

### 2.2. Participants and Sampling Procedures

Out of a total of 450 patients, with a margin of error of 5%, a 95% confidence level, and a response distribution of 50%, 365 participants initially responded to the study. However, 66 participants provided incomplete answers, and 90 did not provide consent to participate in the study. As a result, they were excluded, leaving 209 participants included in the final analysis. The high dropout rate, with 66 participants providing incomplete responses and 90 refusing consent, resulted from several factors. Incomplete answers may have stemmed from time constraints or disengagement during interviews. Consent refusal may have been due to privacy concerns, a lack of interest, or hesitation to participate. The study’s medical details or required commitment may have also led some to opt out.

Participants were required to be between 18 and 65 years of age, to have hypertension, to reside in the Abha Asir region of Saudi Arabia, and to be willing to provide informed consent. The study excluded pregnant women, individuals with psychiatric disorders, those with critical illnesses, and hypertensive patients who were unable to communicate. Cases with missing data were immediately excluded from the analysis. Participants were selected using simple random sampling from the community center. According to the Saudi Hypertension Management Society (SHMS), a digital automated or mercury sphygmomanometer is used to diagnose hypertension if the systolic blood pressure (SBP) is greater than 140 mmHg and the diastolic blood pressure (DBP) is greater than 90 mmHg [17].

### 2.3. Data Collection Method and Tools

The structured questionnaire for this study was prepared using Google Forms, and the link to the form was used during face-to-face interviews. While conducting the interviews, participants’ responses were directly entered into the Google Form, which allowed for easier data extraction for analysis. The data for this study were collected through a questionnaire. Section 1 includes information on demographic and sociodemographic factors, such as gender, occupation, age ranges, marital status, education level, the place of residence, and health insurance status. It also covers family income, social habits (e.g., smoking), body mass index, the use of blood pressure medication, past medical history, physical activity, and salt restriction practices. Section 2 contains the 36-Item Short Form Survey (SF-36) questions, covering eight domains, to assess health-related quality of life (HRQoL). The SF-36 is a widely used self-administered instrument in HRQoL research [18], and its Arabic version was used in this study [19]. The SF-36 consists of eight domains: Physical Functioning (PF), which uses 10 items to assess health conditions that affect normal physical activity; Role Limitations Due to Physical Health (RP), with 4 items measuring functional limitations caused by physical health issues; Vitality Energy/Fatigue (VT), consisting of 4 items, to evaluate the participants’ levels of energy and fatigue; Body Pain (BP), consisting of 2 items that evaluate pain severity and its impact on daily activities; General Health (GH), including 5 items that assess individuals’ perceptions of their health and its trends over time; Social Functioning (SF), with 2 items; Role Limitations Due to Emotional Problems (RE), measured by 3 items that evaluate functional limitations caused by emotional issues; and Emotional Well-being/Mental Health (MH), which uses 5 items to assess emotional well-being. Reliability, as assessed using the Cronbach’s alpha coefficient, was 0.931 for Physical Functioning (PF), 0.841 for Role Limitations Due to Physical Health (RP), 0.801 for Role Limitations Due to Emotional Problems (RE), 0.685 for Vitality Energy/Fatigue (VT), 0.703 for Emotional Well-being/Mental Health (MH), 0.654 for Social Functioning (SF), 0.778 for Bodily Pain (BP), and 0.720 for General Health (GH), indicating good internal consistency. The SF-36 items are usually evaluated using a Likert scale, with 1 denoting not at all, 2 a little, 3 moderately, 4 quite a bit, and 5 extremely. The eight domains were categorized into two summary components: the Physical Component Summary (PCS), which includes Physical Functioning, Role Limitations Due to Physical Health, Bodily Pain, and General Health, and the Mental Component Summary (MCS), which includes Vitality Energy/Fatigue, Social Functioning, Role Limitations Due to Emotional Problems, and Emotional Well-being/Mental Health. The items that did not include Reported Health Transition were coded and converted into a scale ranging from zero (worst quality of life) to 100 (best quality of life) [20].

### 2.4. Ethical Approval

The method was accepted by King Khalid University’s institutional review board (ECM No. 2024-3145). All work was performed in accordance with the relevant guidelines and regulations. On the cover page of the questionnaire, a consent statement was inserted, stating that they would be permitted to continue if they agreed to participate in the study. All information was kept private and utilized exclusively for academic purposes.

### 2.5. Statistical Analysis

IBM SPSS Statistics, version 20 for Windows, was used to perform the statistical analysis. Data were analyzed and coded. Frequencies and percentages were used to summarize the research participants’ variables. A multiple linear regression analysis was conducted to investigate the association between the independent variables and the SF-36 domains. A *p*-value of less than 0.05 was considered statistically significant.

## 3. Results

The sample consisted of 209 hypertensive patients, with a higher number of females 122 (58.4%) than males (87 (41.6%). The majority of the participants were employed, 121 (57.9%), and the age distribution showed 79 (37.8%) in the 18 to 30 years age group, 85 (40.7%) in the 31 to 50 years age group, 22 (10.5%) in the 51 to 60 years age group, and 23 (11.0%) aged over 60. Most participants were married, 120 (57.4%), and the majority had completed university education, 131 (62.7%). In terms of monthly family income, 67 (32.1%) had an income between 5000 and 9999 Saudi Riyals (SAR). See Table 1.

A small proportion of participants (N = 30, 14.4%) were smokers, while the majority (N = 179, 85.6%) did not smoke. In terms of medication use, (N = 106, 50.7%) participants were using one blood pressure drug, (N = 41, 19.6%) were using two or more, and (N = 62, 29.7%) were not on any antihypertensive medication. Past medical history/comorbidities: A total of (N = 20, 9.6%) had COPD, (N = 52, 24.9%) had diabetes, (N = 61, 29.2%) had dyslipidemia, (N = 10, 4.8%) had a history of stroke, and (N = 26, 12.4%) had cardiovascular disease. Physical exercise: A total of (N = 94, 45%) participants engaged in physical exercise. Salt restriction: A total of (N = 90, 43.1%) participants reported following salt restrictions. (See Table 2.)

The SF-36 health survey results for the hypertensive population indicate moderate levels of health and well-being challenges. The Physical Functioning score (58.75 ± 34.37) suggests common physical limitations, with significant variability in how these limitations affect daily activities. Role Limitations Due to Physical Health (62.91 ± 39.76) and Role Limitations Due to Emotional Problems (66.98 ± 39.62) show moderate interference with daily roles, with emotional issues having less impact than physical health concerns. The Vitality Energy/Fatigue score (51.77 ± 18.02) indicates moderate fatigue and low energy, while Emotional Well-being/Mental Health (57.85 ± 19.39) reflects a range of emotional health, from distress to better well-being. Social Functioning (65.07 ± 25.88) is moderately affected by hypertension, with some experiencing social limitations, and Bodily Pain (65.99 ± 27.63) reveals a broad spectrum of discomfort among participants. Finally, the General Health score (64.06 ± 17.77) suggests slightly above-average health perceptions, though with considerable variability, reflecting differing experiences of overall health among the patients. These findings highlight the diverse impacts of hypertension on physical, emotional, and social aspects of life. See Table 3.

Physical Component Summary (SF-36): The domains were analyzed using multiple linear regression. Age (31–50 years) is associated with better general health perceptions (B = 8.086, *p* = 0.017). Smoking is associated with better physical functioning (B = 13.615, *p* = 0.046). The number of BP medications is linked to worse physical functioning (B = −18.631, *p* = 0.010) and lower general health perceptions (B = −8.196, *p* = 0.036). Complications show significant negative effects on Physical Functioning, role limitations, Bodily Pain, and General Health perceptions (*p* < 0.05). Exercise reduces Bodily Pain (B = −8.487, *p* = 0.038). Salt Restriction improves Physical Functioning (B = 12.339, *p* = 0.008) but has no significant effects on other outcomes. See Table 4.

Mental Component Summary (SF-36): The domains were analyzed using multiple linear regression. Income (SAR 5000–9999) was significantly associated with higher vitality (B = 7.632, *p* = 0.038) and with social functioning (B = 16.465, *p* = 0.035). The number of complications showed a significant negative impact on Vitality Energy/Fatigue (B = −2.847, *p* = 0.046), Social Functioning (B = −4.804, *p* = 0.015), and Emotional Well-being/Mental Health (B = −5.075, *p* = 0.001). The no exercise group showed significantly lower Vitality Energy/Fatigue (B = −7.720, *p* = 0.004) and Social Functioning (B = −8.781, *p* = 0.018). Married individuals had significantly better Emotional Well-being/Mental Health (B = 8.947, *p* = 0.006). The age group 51–60 years had higher Social Functioning (B = 16.681, *p* = 0.023). Uneducated individuals had significantly lower Social Functioning (B = −24.541, *p* = 0.003). Income (SAR 15,000) was associated with significantly lower Social Functioning (B = −12.323, *p* = 0.022). See Table 5.

## 4. Discussion

This study explores HRQoL among adult patients with hypertension, emphasizing socio-demographic, economic, and clinical features. The sample of 209 participants, predominantly female (58.4%), reflects higher hypertension prevalence or better diagnosis in women. The significant impact of hypertension on health-related quality of life is underscored by the SF-36 health survey. Patients with hypertension exhibited reduced mean scores across all domains of HRQoL. In comparison to studies conducted in Ethiopia [21], Vietnam [22], Iran [23], and Nigeria [24], the mean scores across all domains of health-related quality of life were higher in our study population. Higher HRQoL scores may reflect better healthcare access, hypertension management, and social support, with variations due to infrastructure, economic status, and cultural factors. Hypertension impacts physical, emotional/mental health, and social well-being, highlighting the need for personalized care [22]. The SF-36 results show lower HRQoL, particularly in Physical Functioning, Vitality Energy/Fatigue, and Emotional Well-being/Mental Health, emphasizing the need for targeted interventions to improve patient outcomes.

The lifestyle factors of the patients in our study indicate that a smaller proportion adhered to salt restriction compared to those in a previous study. More patients in our study engaged in physical exercise, while fewer reported smoking, in contrast to the other study [21]. Reduced bodily pain was significantly correlated with physical activity in the physical component of HRQoL domains. In the mental component, a lack of exercise was linked to reduced vitality and social functioning. Sedentary individuals reported poorer HRQoL compared to those who exercised, consistent with findings from a Vietnamese study [22]. This may be attributed to higher resting heart rates in inactive individuals, which strain the heart and arteries [25]. Regular exercise enhances well-being and reduces hypertension risks. The positive effects on HRQoL may stem from improved social interactions, outdoor activities, increased self-esteem, positive self-perceptions, or biological mechanisms like elevated endorphin levels [21,26]. The health-related quality of life of patients with hypertension can be improved through the, promotion of a healthy lifestyle. Regular exercise has positive effects on patients’ social and physical functioning, which aligns with prior research [27,28].

This study identifies factors affecting physical health in lower-income populations. The findings of this study emphasize the complex interplay of age, education, health conditions, and lifestyle factors in determining physical health. Targeted interventions that address these factors can play a critical role in improving quality of life and reducing health disparities in lower-income populations. Addressing comorbidities, promoting health education, and supporting lifestyle changes can mitigate disparities and improve outcomes. Studies have shown that among Korean American patients with chronic conditions such as hypertension, lower levels of social support are associated with poorer HRQoL [29]. Family support has been identified as a significant factor influencing treatment adherence for hypertension [30]. Additionally, emotional support has been found to positively impact HRQoL in these patients [31]. In our study, the presence of comorbidities, a low level of education and being single were linked to poorer vitality and social functioning. According to this study, patients with comorbidities scored lower on HRQoL physical and environmental domains, consistent with research from Mekelle and Jimma [32,33]. The early diagnosis and treatment of chronic diseases are essential to improving quality of life for hypertensive patients with comorbidities. Patients with hypertension experience a lower quality of life when they have co-occurring clinical disorders [34].

In the present study, vitality was positively associated with social functioning, and middle-income patients reported better vitality and social functioning, while higher-income patients showed lower social functioning. According to earlier research conducted in China [35], South Korea [36], and Pakistan [37], individuals with hypertension from lower-income groups have lower HRQoL. Another study found that low income was associated with reduced scores for vitality and mental health among patients [35]. In our study, the 31–50 age group reported better general health. Research from multiple countries shows that aging negatively impacts physical HRQoL [24,38,39,40,41,42] due to accumulated cellular damage, reduced physiological function, and increased disease susceptibility [43]. Policies aimed at improving health literacy and access to healthcare services are essential for supporting these efforts and ensuring that all individuals, regardless of income, can achieve optimal physical health. In our study, married individuals had better mental health. This finding is consistent with another study showing that the lower HRQoL in single individuals may be attributed to mental health challenges, increased loneliness, and the potential for social isolation over time [44]. These results suggest the importance of prioritizing psychological support for these patients, both in clinical settings and within the community. The study results show that, for individuals aged 18 to 65 years, interventions focusing on physical activity, health literacy, and social support are effective in improving health-related quality of life (HRQoL) and hypertension management, while promoting physical and mental well-being, self-management, and long-term health outcomes.

The SF-36 findings reveal that age, income, and marital status influence health outcomes in patients with hypertension, with complications and BP medications negatively affecting physical and mental health. Salt restriction improved physical functioning, and exercise reduced body pain. These results emphasize the need for interventions targeting complications, lifestyle, and psychological well-being to enhance HRQoL in this population.

### Limitations

The study has limitations that can be addressed in future research. In a cross-sectional study, outcome (e.g., SF-36 health-related quality of life) and predictor variables (sociodemographic, economic, and clinical features) are measured simultaneously, making causality unclear. It is uncertain whether hypertension directly affects quality of life or if other factors influence both, requiring longitudinal or experimental studies for causal inference. Despite face-to-face data collection, response bias may have occurred due to social desirability or recall limitations. Additionally, selection bias could be present if certain groups were more likely to participate or provide complete responses. This study focused on a single region due to practical and logistical constraints, such as limited resources and time. Conducting the research in one region allowed for a more controlled environment and consistent data collection. However, this decision limits the generalizability of the findings, as the results may not fully represent individuals from other regions or diverse populations with different sociodemographic, economic, and clinical characteristics.

## 5. Conclusions

This study highlights the significant impact of sociodemographic, economic, and clinical features on the health-related quality of life (HRQoL) of hypertensive patients, with low scores observed across all domains. Key determinants include age, income, marital status, and comorbidities. The lifestyle factors of the patients in our study indicate that a smaller proportion adhered to salt restriction compared to those in a previous study. Reduced bodily pain was significantly correlated with physical activity in the physical component of HRQoL domains, while in the mental component, a lack of exercise was linked to reduced vitality and social functioning. Promoting healthy lifestyle changes, such as exercise and salt restriction, can enhance physical functioning and reduce body pain. Addressing complications, improving health literacy, and providing psychological support are vital, particularly for individuals with limited social support or co-existing conditions. Targeted interventions should integrate clinical management (e.g., blood pressure control and comorbidity treatment) with psychosocial support (e.g., stress reduction and social assistance) to enhance well-being. This is especially crucial for lower-income populations, who often face healthcare access barriers, financial constraints, and higher stress levels. Policies promoting affordable healthcare, lifestyle interventions, and community-based support can significantly improve their HRQoL. Tailoring health intervention strategies to specific contexts is essential for enhancing the HRQoL of patients with hypertension.

## Figures and Tables

**Table 1:** Demographic and socioeconomic characteristics of hypertensive patients.

Characteristics	Categories	Number (%)
Gender	Male	87 (41.6)
	Female	122 (58.4)
Occupation	Employed	121 (57.9)
	Unemployed	88 (42.1)
Age groups	18 to 30	79 (37.8)
	31–50	85 (40.7)
	51–60	22 (10.5)
	Above 60 years	23 (11.0)
Marital status	Yes	120 (57.4)
	No	89 (42.6)
Level of Education	Primary level	15 (7.2)
	Secondary level	52 (24.9)
	University level	131 (62.7)
	Other (Uneducated)	11 (5.3)
Monthly Family Income	SAR <5000	52 (24.9)
	SAR 5000 to 9999	67 (32.1)
	SAR 10,000 to 14,999	42 (20.1)
	Above SAR 15,000	48 (23.0)

**Table 2:** Clinical and lifestyle factors of hypertensive population.

Variables	Categories	Number (%)
**Smoking**	Yes	30 (14.4)
	No	179 (85.6)
**Number of blood pressure drugs used**	Only one	106 (50.7)
	Two or more	41(19.6)
	None	62 (29.7)
**Past medical history/Comorbidities**	Yes	
COPD	20 (9.6)	
Diabetes	52 (24.9)	
Dyslipidemia	61 (29.2)	
Stroke	10 (4.8)	
Cardiovascular disease (myocardial infarction and angina pectoris)	26 (12.4)	
**Physical exercise**	94 (45)	
**Salt restriction**	90 (43.1)	

**Table 3:** Means and standard deviations for the SF-36 HRQoL questionnaire domains.

SF-36 Domains	Mean ± SD
Physical Functioning	58.75 ± 34.37
Role Limitations Due to Physical Health	62.91 ± 39.76
Role Limitations Due to Emotional Problems	66.98 ± 39.62
Vitality Energy/Fatigue	51.77 ± 18.02
Emotional Well-being/Mental Health	57.85 ± 19.39
Social Functioning	65.07 ± 25.88
Bodily Pain	65.99 ± 27.63
General Health	64.06 ± 17.77

SD: standard deviation.

**Table 4:** Physical Component Summary (SF-36)—multiple linear regression analysis of independent variables versus dependent variables.

Variables	Physical Functioning			Role Limitations Due to Physical Health			Bodily Pain			General Health Perceptions		
	B	t	*p* Value	B (SE)	t	*p* Value	B (SE)	t	*p* Value	B (SE)	t	*p* Value
Gender—male	Reference											
Female	−2.792	−0.548	0.584	3.333	0.553	0.581	0.771	0.178	0.859	−1.425	−0.516	0.607
Occupation												
Working	Reference											
Not working	−7.257	−1.369	0.172	−8.501	−1.357	0.176	1.473	0.327	0.744	4.069	1.416	0.159
Age group												
18–30 years	Reference											
31–50 years	7.393	1.192	0.235	11.719	1.599	0.111	7.312	1.387	0.167	8.086	2.404	0.017
51–60 years	9.559	1.013	0.312	8.853	0.794	0.428	−0.044	−0.005	0.996	2.864	0.560	0.576
>60 years	9.938	0.885	0.377	5.399	0.407	0.684	−10.340	−1.083	0.280	3.207	0.527	0.599
Marital status												
Unmarried	Reference											
Married	−0.106	−0.019	0.985	7.433	1.143	0.254	3.391	0.725	0.470	1.743	0.584	0.560
Education—university	Reference											
Primary	−12.202	−1.139	0.256	−2.068	−0.163	0.870	1.570	0.172	0.863	−3.088	−0.531	0.596
Secondary	3.316	0.565	0.573	12.653	1.823	0.070	−1.392	−0.279	0.781	−3.080	−0.967	0.335
Uneducated	−11.421	−1.067	0.287	−9.630	−0.761	0.448	−5.673	−0.623	0.534	−5.484	−0.944	0.346
Income												
SAR <5000	Reference											
SAR 5000–9999	6.992	1.073	0.285	4.353	0.565	0.573	5.573	1.005	0.316	5.108	1.445	0.150
SAR 10,000 to 14,999	8.326	1.106	0.270	−5.441	−0.612	0.541	−2.266	−0.354	0.724	−0.433	−0.106	0.916
SAR 15,000	−10.948	−1.587	0.114	−15.595	−1.913	0.057	0.714	0.122	0.903	1.248	0.334	0.739
Smoking status												
No	Reference											
Yes	13.615	2.005	0.046	12.838	1.600	0.111	6.997	1.211	0.227	0.655	0.178	0.859
Number of BP drugs used												
Only one	Reference											
Two or more	−18.631	−2.606	0.010	6.357	0.752	0.453	−7.924	−1.303	0.194	−8.196	−2.113	0.036
None	2.449	0.482	0.630	11.649	1.940	0.054	0.207	0.048	0.962	−3.960	−1.437	0.152
No. of comorbidities	−5.940	−2.342	0.020	−13.232	−4.415	0.000	−4.979	−2.309	0.022	−4.290	−3.119	0.002
Exercise												
Yes	Reference											
No	2.669	0.558	0.577	−5.120	−0.906	0.366	−8.487	−2.088	0.038	−3.544	−1.367	0.173
Salt restriction												
Yes	Reference											
No	12.339	2.678	0.008	8.800	1.616	0.108	1.355	0.346	0.730	−1.246	−0.499	0.619

B: beta value, SE: standard error, *p* < 0.05 significant.

**Table 5:** Mental Component Summary (SF-36)—multiple linear regression analysis of independent variables versus dependent variables.

Variables	Vitality Energy/Fatigue			Social Functioning			Role Limitations Due to Emotional Problems			Emotional Well−Being/Mental Health		
	B	t	*p* Value	B	t	*p* Value	B	t	*p* Value	B	t	*p* Value
Gender												
Male	Reference											
Female	2.621	0.918	0.360	−0.561	−0.143	0.887	−6.183	−1.022	0.308	4.853	1.631	0.105
Occupation												
Working	Reference											
Not working	−1.993	−0.672	0.503	2.051	0.501	0.617	0.944	0.150	0.881	−1.880	−0.607	0.544
Age group—18–30 years	Reference											
31–50 years	3.753	1.081	0.281	12.568	2.626	0.009	16.463	2.237	0.026	4.590	1.267	0.207
51–60 years	2.421	0.458	0.647	16.681	2.291	0.023	17.858	1.595	0.112	7.318	1.328	0.186
>60 years	−2.450	−0.390	0.697	16.553	1.910	0.058	17.358	1.303	0.194	9.632	1.469	0.143
Marital status												
Unmarried	Reference											
Married	5.774	1.874	0.063	−1.796	−0.423	0.673	−0.507	−0.078	0.938	8.947	2.783	0.006
Education												
University	Reference											
Primary	−2.696	−0.449	0.654	−10.144	−1.227	0.221	−0.076	−0.006	0.995	−5.209	−0.832	0.406
Secondary	3.317	1.008	0.315	−4.287	−0.946	0.346	7.312	1.049	0.296	−4.513	−1.315	0.190
Uneducated	−2.773	−0.462	0.644	−24.541	−2.969	0.003	−15.062	−1.185	0.238	−6.659	−1.064	0.288
Income												
SAR <5000	Reference											
SAR 5000–9999	7.632	2.090	0.038	−1.246	−0.248	0.805	16.465	2.128	0.035	10.213	2.682	0.008
SAR 10,000–14,999	2.831	0.671	0.503	0.693	0.119	0.905	8.817	0.987	0.325	4.410	1.003	0.317
SAR 15,000	1.117	0.289	0.773	−12.323	−2.314	0.022	−7.691	−0.939	0.349	2.686	0.667	0.506
Smoking Status												
No	Reference											
Smoking	1.226	0.322	0.748	8.402	1.603	0.111	−4.960	−0.615	0.539	−1.719	−0.433	0.665
Number of BP drugs used												
Only one	Reference											
Two or more	−0.424	−0.106	0.916	−8.539	−1.547	0.124	−12.358	−1.456	0.147	−4.311	−1.032	0.303
None	−2.607	−0.916	0.361	9.133	2.328	0.021	4.355	0.722	0.471	0.631	0.212	0.832
No. of comorbidities	−2.847	−2.004	0.046	−4.804	−2.454	0.015	−11.983	−3.981	0.000	−5.075	−3.426	0.001
Exercise												
Yes	Reference											
No	−7.720	−2.883	0.004	−8.781	−2.380	0.018	−9.456	−1.667	0.097	−1.261	−0.452	0.652
Salt restriction												
Yes	Reference											
No	−0.018	−0.007	0.994	4.882	1.373	0.172	5.977	1.093	0.276	−1.019	−0.379	0.705

B: beta value, SE: standard error, *p* < 0.05 significant.

## Data Availability

The original contributions presented in the study are included in the article; further inquiries can be directed to the corresponding author.
